# Pruning Decision Rules by Reduct-Based Weighting and Ranking of Features

**DOI:** 10.3390/e24111602

**Published:** 2022-11-03

**Authors:** Urszula Stańczyk

**Affiliations:** Department of Computer Graphics, Vision and Digital Systems, Silesian University of Technology, Akademicka 2A, 44-100 Gliwice, Poland; urszula.stanczyk@polsl.pl

**Keywords:** decision rule, rough set theory, reduct, attribute weighting, ranking, rule pruning

## Abstract

Methods and techniques of feature selection support expert domain knowledge in the search for attributes, which are the most important for a task. These approaches can also be used in the process of closer tailoring of the obtained solutions when dimensionality reduction is aimed not only at variables but also at learners. The paper reports on research where attribute rankings were employed to filter induced decision rules. The rankings were constructed through the proposed weighting factor based on the concept of decision reducts—a feature reduction mechanism embedded in the rough set theory. Classical rough sets operate only in discrete input space by indiscernibility relation. Replacing it with dominance enables processing real-valued data. Decision reducts were found for both numeric and discrete attributes, transformed by selected discretisation approaches. The calculated ranking scores were used to control the selection of decision rules. The performance of the resulting rule classifiers was observed for the entire range of rejected variables, for decision rules with conditions on continuous values, discretised conditions, and also inferred from discrete data. The predictive powers were analysed and compared to detect existing trends. The experiments show that for all variants of the rule sets, not only was dimensionality reduction possible, but also predictions were improved, which validated the proposed methodology.

## 1. Introduction

Stylometry is a study of writing styles that exploits not qualitative but quantitative descriptors, postulating the uniqueness of linguistic traits characteristic for authors [1]. Researchers in this domain agree that given access to a sufficient number of representative samples of writing, it is possible to construct a stylistic profile. This profile, expressed in measurable terms called style-markers (or *writer* or *author prints*) [2], can then be approximated with a certain degree of reliability. This can be used for purposes of linguistic characterisation, comparison of writing styles, or, which is widely perceived as the most important task, to perform *author attribution* [3], which denotes recognition of the authorship for texts when it is either unknown or questioned [4,5].

This human individuality, expressed through linguistic preferences and stylistic habits, is further evidenced by the very wide variety of features that can be used as style descriptors. In their nature, they can be nominal and categorical, discrete but numeric and ordinal, or continuous. The diversity of the offered markers can be considered both as an advantage and a disadvantage. With more attributes, more options are open and there can be deeper insight into the knowledge domain, but at the same time, the choice of a particular set of features for any given task is not trivial [6]. The process of attribute selection can be supported by approaches dedicated to the estimation of the importance of features, such as the calculation of rankings, and also data-mining techniques, employed in stylometric tasks, offer their own dimensionality reduction mechanisms [7].

Stylometric data exploration typically involves either calculations referring to statistics or machine learning algorithms [8,9]. For the former approach, models for probabilities of specific transitions can be constructed for texts and then compared, or distributions of certain characteristics of a text can be plotted and contrasted [10,11]. A task of stylometric analysis can be treated as a pattern recognition problem, with patterns present in the textual data to be detected and then recognised by methods from the artificial intelligence domain, such as, for example, artificial neural networks. When knowledge mined from data is presented in a form that is directly accessible and relatively simple to interpret, it brings enhanced understanding of the stylometric domain, and the decision rules inferred from the data samples provide such opportunity by their transparent structure.

The rough set theory was invented to support operation on uncertain and incomplete knowledge [12]. The Classical Rough Set Approach (CRSA) enables the observation of the presence or absence of features through a fundamental relation of indiscernibility. This allows only nominal classification with an induction of decision rules based on the notions of decision reducts and approximations of concepts [13]. When objects of the universe are described with numeric and ordinal attributes, either some discretisation transformation needs to be employed [14,15], or a modification of the classical rough exploration can be used, such as the Dominance-Based Rough Set Approach (DRSA) [16]. In DRSA processing, dominance relation replaces indiscernibility, and then also the observation of preference orders and ordinal classification become possible.

The paper presents research focused on the stylometric task of authorship attribution, establishing the importance of features used to describe authorial profiles and the influence of this importance on the performance of constructed rule classifiers. The relevance of attributes was studied in the original continuous domain and also after discretisation transformations, and it was perceived through constructed rankings. As a basis for the rankings, the proposed weighting factor was used, assigning scores referring to sets of decision reducts found for both numeric and discretised input data. For all variants of data, before and after discretisation, decision rules were inferred using correspondingly either DRSA or CRSA. The sets of decision rules were then pruned while governed by rankings, discarding rules with conditions on rejected attributes. Three versions of decision algorithms were processed: with continuous conditions, with conditions induced from numeric attributes and then discretised, and with conditions inferred from discrete data. Performed experiments show that for all studied sets of rules, their performance in labelling unknown samples could be improved at some point of the process of rule pruning, providing dimensionality reduction and higher predictive powers, which validated the proposed methodology.

The main contributions of the research presented in this paper include:

- Comparison of properties for sets of decision reducts found for the same input data space seen through various perspectives, in the continuous domain as well as discrete, with selected discretisation approaches employed;
- Construction of attributes rankings based on the proposed weighting factor referring to generated sets of reducts;
- Application of reduct-based attribute rankings in the process of pruning decision rules inferred from continuous data, with conditions on continuous values and discretised conditions, and for rules induced from discretised data;
- Observation of trends in performance for various variants of rule classifiers in the perspective of ranking-driven attribute and rule selection.

The structure of this paper is organised as follows. Section 2 contains descriptions for elements of the theoretical background. Section 3 provides an explanation for all steps of the proposed research methodology, while in Section 4, the performed experiments are detailed, with discussion of the results obtained. Concluding remarks and indication of potential future research paths are given in Section 5.

## 2. Preliminaries

This section presents fundamental issues under consideration in the described research works, such as the stylometric domain of textual analysis and characteristics of stylometric features, approaches leading to establishing importance of characteristic features, discretisation algorithms, rough set processing, and indicators of rule quality used for their selection.

### 2.1. Nature of the Stylometric Input Space

The individuality and uniqueness of writing styles is the fundamental assumption of stylometry [17]. At its origin, the stylometric processing of texts involved tedious comparative analysis performed by scholars looking for more striking phrases, specific collocations. With the advent of word processors and easy access to various sources, imitation or even the falsification of someone else’s style became less difficult, and textual analysis took advantage of high computational powers of computers. Instead of searching for obvious tells, more subtle linguistic descriptors are studied to find *authorial invariants*, which are such sets of features that enable the characterisation of writing profiles for authors and lead to an approximation of styles [18]. This allows for comparison of elements of styles for various authors, and for authorship attribution, which is considered as the most important of stylometric tasks.

The elements that are studied can vary significantly in scale [19], and for observed markers, statistics can be calculated, such as frequencies of occurrence, averages, distributions, and matrices of transitions [10,20]. Such detailed analysis allows one to catch individual preferences and habits of writing, which are so ingrained that they are used to some extent without conscious thought; therefore, they are much less likely to be forged. *Lexical* descriptors can start at the lowest level of single letters and other characters, or with their groups, words, phrases, sentences, or whole paragraphs. *Syntactic* markers track the patterns of sentence construction, whether they are simple or with complex conditional clauses, through employed punctuation marks [21]. *Structural* style-markers focus on the general text structure, its units, their arrangement, along with formatting, layout. Features from the *content-specific* category consider words or phrases of special importance, with specific meaning in some context.

To build an author profile, several examples of writing need to be analysed; the more there are, the closer the approximation of writing style. The choice of stylometric markers to be employed for a task depends on types and, in particular, on the lengths of these text samples, as for shorter forms, different rules apply. Different text genres also determine a different selection of style markers. Once the profile is constructed, for text samples of unknown or questioned authorship, the same characteristics are obtained and then compared; thus, the task of attributing a text to some author becomes a problem of supervised learning. Samples of known authorship are labelled with their authors, and for those whose authorship is unknown, labels are predicted based on knowledge of patterns discovered in stylometric data mining [22].

The methods used in stylometric processing typically refer to either statistics or techniques and methods from the artificial intelligence domain [23]. In the former case, for example, detailed language models can be built for texts, observing transitions between letters and characters, and then, Markov chains can be employed [24]. In the latter, artificial neural networks can be trained on characteristics of training text samples and then assign authors for test instances [25], or decision rules can be inferred from data [26]. This last approach has the advantage of easy access to patterns discovered in data exploration, which was why this type of learner was chosen as the one to be used in the described research.

### 2.2. Importance of Characteristic Features

To learn the importance of features that describe observed concepts [27], expert domain knowledge is the first and primary source to be referred to. Unfortunately, this source is quite often overflowing with to some extent conflicting information. Accounts of efficiency based on various research can be contradictory or simply insufficient, as the relevance of employed attributes depends on many factors, and it should always be studied with caution. In addition, attention should be paid to particular context and research constraints as well as the experimental setup. This is one more reason for the great popularity of feature selection and reduction algorithms and methods, which, through various processing, support the task of finding some advantageous set of features for a given problem [28].

*Ranking* is one of the mechanisms dedicated to ordering variables according to their importance, however this importance is defined or perceived. Some algorithms return only ranking positions, without assigning any particular score, for example by selecting attributes in the sequential search process, executed either forward or backward [29]. In the former case, processing starts with the empty set to which the variables are next added, either one by one or in groups, while for the latter, the complete set of features is available at the starting point, and then, some of variables are gradually discarded. The feature selection can be stopped once some requirements are met, such as the resulting number of variables, or the importance of these attributes being above or below some set threshold.

When a score is given for features [30], the algorithms divide themselves into two categories. The first group studies all variables and always returns non-zero rank. All variables are considered as relevant but of course with observed degrees of relevance. Relief and its variants are popular examples of this category of methods [31]. The second group of approaches can consider some features as completely irrelevant and assign to them the rank of zero. This can be achieved through referring to notions from information theory, such as entropy, and then in calculations, for example, information gain or gain ratio can be obtained. Such irrelevant attributes can then be rejected together, without any distinction between their importance.

Feature selection methods can operate without the support of any learner, and then, they are called *filters*. *Wrappers* are built around a specific inducer that points to specific features, which are seen as most advantageous. *Embedded* algorithms are based on mechanisms inseparable from a learner, and they are a part of it. However, these broad categories can be mixed; that is, it is possible to use a wrapper as a filter when the ranking of attributes is returned and employed by a different inducer. Alternatively, an embedded mechanism, inherent to some method of data exploration, can be exploited for the purpose of weighting features [32]. In the research presented, such a mechanism, namely decision reducts, specific to the rough set theory, were used to define the weighting factor for attributes.

### 2.3. Discretisation Approaches

Discretisation is a transformation process applied to input features, which changes the representation of their values from continuous to categorical. For the entire range of values for a variable, a certain number of intervals (also called *bins*) is constructed. Not all data exploration methods are capable of mining knowledge from numeric attributes, and even when they do, they can benefit from the change of representation, so discretisation is called an enabling technique [33]. The transformation can be perceived as some simplification, loss of unnecessary detail, or attempt at removing noise. Typically, at the same time some loss of information can be observed; therefore, discretisation should always be treated with caution [34].

Discretisation starts with the ordering all distinctive values of an attribute, detecting minimum and maximum, and then, cut-points are selected that are supposed to divide the range of values into some number of bins. The number of intervals can be either requested in parametric methods or result from calculations specific to transformation algorithms. In the latter case, the methods often refer to the Minimum Description Length (MDL) Principle and the calculation of entropy when considered with respect to the original range of values before and after division into bins. The two popular algorithms in this category are *supervised* discretisation approaches proposed by Fayyad and Irani [35] and Kononenko [36]. Supervised transformation means taking into account class labels for samples at the time of cut-point selection. Candidate cut-points are evaluated, and the ones that are chosen in the best way support recognised classes. Both algorithms work in the top–down direction. They begin by assigning a single interval to represent all values of a transformed variable, and then, they try to split this interval in the iterative process executed until the stopping criterion is met. When none of candidate cut-points meets requirements, then this one bin is the sole representative for the attribute, which effectively means that this variable is excluded from considerations in the discrete domain, as its value is then constant for all samples.

In *unsupervised* discretisation, class information is disregarded, and cut-points are decided only based on attribute values. The simplest of methods in this category is *equal width* binning, which can be seen as just a decrease of scale in the input space. The distribution of existing data points is completely disregarded, which is considered to be a huge drawback of this approach, whereas the intervals are constructed in the predefined number to be equal and regularly distributed in space. *Equal frequency* binning takes the distribution of points into account and groups them into the desired number of bins to make the number of instances per bin equal. As a consequence, the intervals are not uniformly distributed; some regions of space can be more densely packed with smaller bins, while in others, there are fewer larger intervals.

Usually, when discretisation procedures are applied, they are a part of the initial pre-processing of data, and only the influence on performance is possibly studied [37,38]. However, the choice of a particular transformation algorithm is not necessarily clear cut, as even for the often-preferred supervised group, there is more than one method to choose from, and testing several approaches is computationally much too demanding. In addition, mining data after discretisation means exploring patterns from which some information was removed. To address both of these considerations, a novel approach was proposed, in which data were explored in the continuous domain to learn patterns that were then stored in induced decision rules. The data and the conditions in the rules were next discretised. Such processing enabled studying the impact of various discretisation methods at significantly lower costs [39]. This proposed methodology was exploited as one of the research paths in the experiments described in the paper, while the other was by standard discretisation followed by data mining.

### 2.4. Rough Set-Based Data Exploration

The universe seen through the rough set perspective is granular [40]. The atoms of knowledge correspond to equivalence classes of objects that cannot be discerned based on values of observed attributes. The relation of *indiscernibility* is one of the fundamental notions of the rough set theory in its classical approach (CRSA), which allows only nominal classification by decision rules inferred from *approximations* of concepts.

The available knowledge is represented by the special form of an information system, which is called a *decision table* (DT). The objects of the universe are described by rows in the decision table, with columns of the table defined by the set of attributes *A*, which consists of a set of *condition* attributes $C=\{c_{1},\ldots ,c_{N}\}$ and a set of decision attributes $D=\{d_{1},\ldots ,d_{M}\}$, A=C⋃D. Although condition attributes are typically numerous, in many classification problems, only single decision attributes are distinguished, D={d}, and its values are simply referred to as *decisions*. Decision rules inferred from DT in their premises list values of condition attributes (such attribute–value pairs are called *descriptors*) that lead to specific decisions in the form given by Equation (1),

(1) $$If\left(c_{i_{1}}=v_{1}\right)\wedge \ldots \wedge \left(c_{i_{k}}=v_{k}\right)then\left(d=v_{d}\right),$$

where $c_{i_{1}},\ldots ,c_{i_{k}}\in C$, values $v_{j}$ belong to domains of condition attributes $V_{c}$ (for each attribute $c_{i}$, $c_{i}:U\to V_{c_{i}}$), and $v_{d}\in V_{d}$.

Through the knowledge represented, the decision table can be said to possess certain descriptive power, but not necessarily all included condition attributes are needed to protect it [13]. If there exists an irreducible subset of attributes that enables the same quality of prediction as the entire decision table, then such a subset is a *relative* or *decision reduct*. In other words, any decision reduct needs to include only such condition attributes that are sufficient to discern all objects in the table with different decisions. The intersection of all reducts is called the *core*, and, when non-empty, it contains such variables that are necessary for classification. The process of looking for decision reducts can be compared to that of search of prime implicants for logic functions, and similarly, if exhaustive, it is very demanding computationally, which is why other algorithms for reduct generation were invented, and heuristic approaches were exploited [41,42].

When the application domain offers features with continuous values, to explore them with the rough set approach in its classic form, firstly, attribute values need to be translated into categorical representation, employing some of the discretisation algorithms. Another way of processing leads through such modification that enables knowledge induction from numeric variables [43]. One of such modifications relies on replacing indiscernibility with the dominance relation [44], resulting in the Dominance-Based Rough Set Approach. In DRSA, the values of all attributes need to be not only ordered, but also preferences for ordering are defined. Then, the conditions on attributes are expressed not by the requested equality, as given in Equation (1), but they are considered as *at least as good* or *at most as good* as the calculated threshold, taking one of the forms provided in Equation (2),

(2) $$\begin{array}{c} If\left(c_{i_{1}}\leq v_{1}\right)\wedge \ldots \wedge \left(c_{i_{k}}\leq v_{k}\right)then\;v_{d}^{\leq }\\ If\left(c_{i_{1}}\geq v_{1}\right)\wedge \ldots \wedge \left(c_{i_{k}}\geq v_{k}\right)then\;v_{d}^{\leq }\\ If\left(c_{i_{1}}\geq v_{1}\right)\wedge \ldots \wedge \left(c_{i_{k}}\geq v_{k}\right)then\;v_{d}^{\geq }\\ If\left(c_{i_{1}}\leq v_{1}\right)\wedge \ldots \wedge \left(c_{i_{k}}\leq v_{k}\right)then\;v_{d}^{\geq } \end{array}$$

In these forms, samples are classified as at most belonging to $v_{d}$ class (top two formulas) or at least belonging to class $v_{d}$ (two bottom forms). In a case of binary classification, the interpretation of such predictions is straightforward; for multiple classes, it could be less intuitive, yet at the same time, the ordinal classification that is executed by DRSA is desirable for problems from a multi-criteria decision-making area.

The induction of decision rules is a widely studied topic [45]. Due to their structures, rule sets offer easy access to knowledge discovered in data mining, enhancing understanding for domains of application, and that is why they are often preferred as inducers. Exhaustive search enables constructing all minimal sets of descriptors leading to particular decisions (for CRSA), or to upward or downward unions of decisions (for DRSA), but such processing has high computational costs. Another path is offered by some heuristics, where, with significantly less effort, only some subsets of rules are inferred, for example, providing a minimal cover of the learning samples. However, with fewer rules, for unknown samples to be labelled in tests, predictions could be at unsatisfactory level due to the lack of coverage, when no rules are found to match the samples. Test samples can constitute some rarer patterns that were not detected in training.

If it can be afforded, then a kind of middle ground among various approaches to rule induction could be found in inferring some larger than necessary set of rules (possibly by exhaustive search) and next performing rule selection. Then, these rules can be chosen that are considered the most interesting ones, however it is defined.

### 2.5. Quality of Rules and Pruning

Regardless of the algorithm used for induction, once a set of decision rules is found, elements of this set can be analysed either individually or as a set [46,47]. In the former case, typically direct characteristics of rules are studied, such as *length* or *support*. The length of a rule gives the number of attribute–value pairs, which are included in the premise part of the rule. Shorter rules are more general and possess better descriptive powers, whereas longer ones provide closer definitions for detected patterns but cause the risk of overfitting. In particular, rows of a decision table could be used directly to construct rules, each containing as many conditions as many attributes were available, yet such an approach would be hardly general and unlikely to result in good predictions. The rule support indicates the number of samples in the training set that were used as a base to infer this rule, or in other words, how many training samples this rule covers. Small values of rule support indicate rare patterns captured by descriptors, while higher values show dependencies observed in many instances, and therefore, they are more likely to appear also in unknown samples to be classified. These two characteristics can be involved in imposing hard constraints on rules to choose only the best.

When treated as a group, rule sets are typically evaluated as any other classifier, that is, by size (cardinality of the set and the number of conditions) and by the fact of how well they can predict classes for previously unseen samples. Then, quality measures refer to aspects of coverage, possibly recall and specificity, in particular for imbalanced classes, when some decision is under-represented while some other is over-represented [48]. For balanced classes and binary classification cases, using classification accuracy seems the most natural, and then, the number of correctly labelled samples, regardless of their labels, to the total number of samples considered, is given.

Still other, more complex quality measures can be defined for rules through the attributes included in their premises, their properties, or combinations thereof [49]. Scores assigned to individual rules can lead to their weighting, and then, rule ranking can be exploited to discard less important rules and keep only some subset of the most advantageous elements. On the other hand, an attribute ranking can be employed for pruning rule sets. Then, from the considered set, the lowest ranking variables are rejected, and along with them, all the rules are discarded that include conditions on these attributes. This last approach shifts the perceived importance of attributes on rules. It was exploited in the reported research, with the goal of observing how the relevance of features, based on decision reducts, is going to influence the performance of rule classifiers constructed in the process of rule pruning, and also considering the impact of data discretisation.

## 3. Proposed Methodology

This section provides an explanation of the experimental setup, with the presentation of all stages of data processing and exploration. For all steps, comments on conditions and constraints are given.

### 3.1. Research Framework

**Figure 1:**
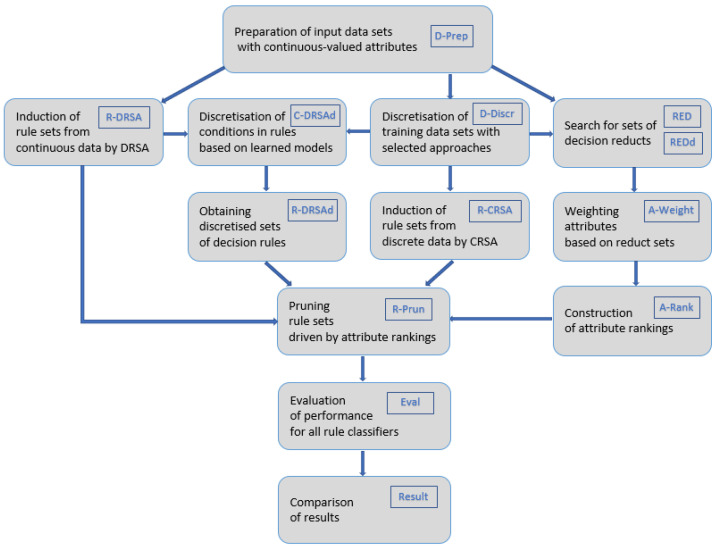
Framework of performed experiments.

The procedure of experiments included several paths and consisted of stages as shown in Figure 1. The starting point was dedicated to the construction of input datasets with numeric attributes (labelled as D-Prep). Next, in one path of exploration, these input data served as a source of knowledge to be mined in order to induce decision reducts. The reducts were inferred while still working in the continuous domain (RED) but also from all variants of input data constructed by discretisation transformation with various approaches (D-Discr), which returned as many sets of reducts (REDd).

All sets of generated decision reducts (RED and REDd) were treated as a new source of available knowledge about attributes [50], and, with the help of the proposed weighting factor, for all variables scores were assigned (A-Weight), which led to their rankings (A-Rank). These rankings were then exploited in the rule filtering process (R-Prun), which was the central meeting point for all research paths, incorporating the results of all of them.

In the research path parallel to the one focused on reducts and attribute weighting and rankings, the input continuous data were mined to induce decision rules with the Dominance-Based Rough Set Approach (R-DRSA). These rule sets constituted one group of rules to be pruned, but they also served as a basis for the construction of the second group. The information on discrete data models found in discretisation of input data (D-Discr) was imposed on continuous conditions present in rules, making them discrete (C-DRSAd), which led to obtaining sets of discretised decision rules (R-DRSAd). The third group of rules for filtering was obtained by application of the Classical Rough Set Approach to discretised data (D-Discr); therefore, the rules (R-CRSA) included conditions in their premises that were discrete from the moment of their construction and did not require any additional processing. These three groups of rule sets, inferred to some extent in parallel paths, were next subjected to pruning.

Rankings originating in decision reducts induced from continuous data (RED) were applied to control rule pruning for both decision rules with conditions in continuous domain (R-DRSA) and their discretised versions (R-DRSAd). On the other hand, for each set of decision rules induced from discrete data (R-CRSA), a corresponding ranking of attributes was used in reduction.

Ranking-driven rule pruning (R-Prun) was executed backwards. Starting with less important attributes, one by one, variables were discarded and with them all the rules from the considered set that included conditions on rejected features. The remaining rules referred only to these attributes that were still retained. All sets and subsets of decision rules were applied as classifiers to unknown samples (Eval), and their performance was studied and compared (Result).

### 3.2. Preparation of Input Datasets

The construction of input datasets started with the selection of authors to be recognised and their works to be processed. Next, text samples were prepared, over which values for the chosen stylometric features were calculated.

Stylometric features selected to approximate writer profiles need to be specific to characterised authors, but since an authorship attribution task involves a comparison of such profiles, also some detectable similarities in writing styles can result in biased recognition. Such common traits are exhibited by writers of the same gender; therefore, authors should be grouped accordingly [51,52]. In the research, two female and two male writers were chosen, namely Edith Wharton and Mary Johnston, and Jack London and James Oliver Curwood, which led to two datasets, correspondingly female writer (F-writers) and male writer (M-writers) datasets.

All four writers are well known for a number of relatively long literary works. These long texts were partitioned into text chunks of sufficient but significantly smaller length to increase the number of available text samples, to allow closer observation of style variations and to make the obtained statistics comparable [53,54]. To avoid using samples that refer to one and the same original work at both the training and the testing stage of data exploration, the groups of text chunks were randomly separated into such two categories.

The frequencies of occurrence for stylometric features were then calculated for the prepared text samples. Lexical markers were employed, referring to the most commonly used function words in the English language, and this set was further supported by syntactic descriptors [21,55], that is, the usage of punctuation marks, resulting together in the set of 100 attributes. In this preliminary set for many variables the calculated values for most samples were found to be equal to zero. Therefore, the set was reduced by applying several ranking algorithms (implemented in WEKA [56] workbench), such as information gain or gain ratio. From the further considerations these features were rejected, which were found as irrelevant by at least one ranking mechanism, and the remaining 24 attributes were only those that were always assigned a non-zero rank. These attributes included 22 lexical and 2 syntactic stylometric descriptors as follows:
afteralmostanyaroundbeforebutbyduringhowneveronsamesuchthatthentherethoughuntilwhatwhetherwhowithin,;

To avoid misunderstanding, when these features were directly referred to in the rest of the paper, in particular in the section commenting on the performed experiments, then the attribute names were given as emphasised (for example: *whether* was found less often in rules than other attributes). Syntactic features were commented on by their names instead of characters, that is, comma, semicolon.

Calculation of frequencies of occurrence means obtaining continuous-valued attributes and the input space in the continuous domain. As a consequence, for exploration of the data, either such learners need to be employed that are capable of dealing with numeric variables, or some discretisation strategy needs to be applied to datasets, or transformation into categorical representation for the discovered patterns should be attempted. In the research described, all three paths were tested. All sets were constructed to constitute cases of binary classification with balanced classes.

### 3.3. Discretisation of Data and Discovered Patterns

Supervised discretisation algorithms are most often preferred as the ones that take into consideration not only attribute values for particular samples but also labels assigned to these samples; thus, they aim at supporting the recognition of classes. In the research works presented in the paper, as representatives of this category, the methods of Fayyad and Irani [35] and Kononenko [36] were used. Both algorithms are non-parametric and return single variants of input datasets (referred to as dsF and dsK, respectively).

From unsupervised discretisation approaches, equal width binning (duw) and equal frequency binning (duf) were employed with some constraints on the input parameter that needs to specify the number of intervals to be constructed to represent ranges of values. For both methods, the input parameter was defined as ranging from 2 to 10 bins, with the step of 1, which resulted in 9 variants of discrete data models (from duw2 to duw10, and from duf2 to duf10). Therefore, for F-writers and M-writers datasets, all together, the number of versions obtained was 21: the single original continuous set, and then 20 discrete sets.

Each version of a dataset included one training and two test sets. With separate sets, the most convenient approach to their discretisation would be through entirely independent processing. It would mean that in the classification process, the comparison of discretisation models between training and testing data would become a part of recognition. It is reasonable to expect some irregularities in the data to be present, and independent transformation could result in the obtaining of not only different cut-points but also possibly different numbers of intervals constructed for the same attributes [57]. To avoid this problem, data samples from test sets were transformed using definitions of ranges constructed for attributes based on training data.

The discretisation employed in the research was used in two ways: as a pre-processing stage of input data, to obtain discrete datasets that were next explored, which is the most popular approach, but also to transform patterns discovered in the continuous domain [39]. This latter methodology is available when a data exploration process returns the mined knowledge in some easily accessible form, such as sets of decision rules. The conditions, listed in the premises of the rules, indicate attribute values leading to specific decisions, and these values were translated to the discrete domain using discretisation models obtained for attributes (denoted as RdsF, RdsK, Rduw*i*, and Rduf*i* correspondingly). With 20 variants of discrete data, there were also 20 variants of discretised rule sets that originally were induced in the continuous domain. Therefore, all together, there were 41 rule sets to be pruned for each dataset: 1 with continuous conditions in rules, 20 with discretised conditions, and 20 with discrete conditions obtained.

### 3.4. Finding Decision Reducts

Decision reducts are a mechanism dedicated to dimensionality reduction, and for the purpose of knowledge representation, their minimal lengths are obviously preferred. Yet, the task of finding a minimal reduct belongs to NP-hard problems, whereas on the other hand, the number of reducts that can potentially be found for a given task with *N* attributes is as high as NN/2. With such high computational costs involved, exhaustive search can be avoided by application of other methods, for example genetic algorithm [58].

In the research, an exhaustive search for reducts was executed for the original data in the continuous domain, with algorithms implemented in the 4eMka software used for DRSA data exploration. For F-writers, this returned 239 decision reducts, with cardinalities ranging from 4 to 12, with one attribute included in all of them (that is, belonging to the core), namely a comma, and also one attribute included in no reduct (*whether*). For the male writer dataset, the number of reducts was much higher, 6072, the core was empty, and all attributes belonged to some reducts.

Since in the research, there were 20 versions of discrete representation of each of the two datasets, the induction of all reducts for all of them would be a very time-consuming task with high computational costs. Therefore, instead, a genetic algorithm was used, which was implemented in the Rough Set Exploration System (RSES) that was exploited for CRSA processing. For this algorithm, the input parameter, specifying the requested number of reducts to be found, was set to 200. However, in two cases, this requirement was not satisfied when it was discovered that the total number of reducts found was lower. It happened for both datasets for unsupervised discretisation through equal width binning with just two bins. In this case, for male writers, 146 reducts could be generated, and for female writers, 178 reducts could be induced.

The sets of decision reducts were treated as the source of information on attributes and through further analysis employed for their ordering and ranking. It was carried out with the help of the proposed weighting factor based on reducts, which is explained in the next section of the paper.

### 3.5. Weighting Factor

Let $S_{Red}=\left\{Red_{1},\ldots ,Red_{P}\right\}$ denote a non-empty set of decision reducts and let *l* denote reduct cardinality or length, with values in the range $\left[l_{min},l_{max}\right]$. The cardinality of a set is returned by card function, so, when applied to a set of reducts, $card(S_{Red})=P$. For the set $S_{Red}$ of reducts and a given condition attribute *c*, let $RED(S_{Red},c)$ be such a subset of decision reducts of the set $S_{Red}$, where the attribute *c* is included in all reducts. Finally, let $RED(S_{Red},c,l)$ denote the subset of reducts selected from $S_{Red}$, all of which contain the given attribute *c*, and all with the specific cardinality *l*.

Based on a set of decision reducts $S_{Red}$, the weighting factor $W_{F}$ for an attribute *c* was defined as follows [59,60]:

(3) $$W_{F}\left(S_{Red},c\right)=\sum_{i=l_{min}}^{l_{max}}\frac{card\left(RED(S_{Red},c,i)\right)}{card(S_{Red})\cdot i}.$$

The values returned by the weighting factor can vary significantly, as it is possible that none of the reducts, all reducts, or some reducts include the given attribute *c*. Reducts can be of the same or varying lengths, and the cardinalities of the sets of reducts depend not only on the data but also on the algorithm employed in the search for reducts. The minimum value of the factor is zero when none of reducts in the set contains the given variable. Therefore, unlike some other ranking mechanisms, such as Relief [31], with the proposed weighting factor, some attributes can be considered irrelevant and assigned the rank of zero. The maximum value would be found for all reducts in the set being of the same length *l*, and all including the attribute. Then, obviously, $l_{min}=l_{max}=l$, and as a consequence $W_{F}=1/l$.

### 3.6. Induction of Decision Rules and Rule Pruning

In the experiments performed for the original form of the input datasets, that is, for numeric attributes, the decision rules were induced once, with the Dominance-Based Rough Set Approach. This type of exploration requires all attributes to be preference ordered. Ordering for continuous-valued variables is natural, preferences are not, as knowledge whether smaller or higher values are more likely to point to some class is something to be learnt from data, and it is not pre-defined. Preferences can be discovered through detailed investigation and close tailoring leading to best rules, but such processing would involve additional costs. Instead, much simpler estimation was obtained. For all attributes, both types of preferences (*cost*: the lower value of an attribute the higher class, or *gain*: the higher attribute value the higher class) were arbitrarily set, and then, minimal cover decision algorithms were generated. The sets of rules with better general properties—higher average supports and lower averaged lengths—were then used to guide selection of preference orders, which were the same for all the attributes in a dataset.

For attributes with decided preference ordering, next, all rules on examples were induced. DRSA processing returned 23,006 decision rules for F-writers and 40,179 rules for M-writers. The lowest supports were equal to 1, meaning that patterns captured by the rules with this support could only be detected in single instances among all included in the training sets. For F-writers, the highest support value was 92, and for M-writers, it was 78. The shortest rules for both datasets contained single descriptors, while the longest included 12 conditions for female writers and 10 for male writers.

Once the datasets were discretised, for all 20 discrete versions per dataset, the decision rules were next inferred by the exhaustive algorithm with CRSA processing. The resulting rule sets greatly varied in cardinalities, from just a few thousand to hundreds of thousands of included rules, and also different properties were evidenced in rule lengths and supports. In the case of the supervised discretisation employed to the data, some variables were found to have single intervals for representation in a discrete domain, which effectively excluded them from further considerations and processing. Then, such attributes were absent in both reducts and rules; therefore, with the assigned zero rank by the weighting factor, they were the first to be rejected.

For the sets of rules, the task of pruning was performed gradually. In each step of processing, a single attribute, selected from a corresponding ranking, was discarded. At the same time, from a set of rules under study, all rules that included conditions on this feature were removed. Rule selection started with the entire sets of induced decision rules and continued until the set remained non-empty, that is possibly until only single variables were left, while all others were discarded. Such processing had the goal of observing general trends; in practical applications, it would be stopped much earlier, when some set criteria were met, because when too few attributes and rules are available, the performance of rule classifiers, when applied for predictions of unknown samples, can become unsatisfactory.

### 3.7. Evaluation of Performance

One of the goals set for the presented research was to observe the influence of various rankings on the selection process of attributes and decision rules. The resulting impact on the performance of rule classifiers was considered as a part of this processing; therefore, some measure of performance needed to be chosen. Depending on the construction of a dataset, in particular when classes are represented in different degrees, when there is some imbalance [61], with multiple classes to be recognised, when some classes are considered as more important than others, or when costs of misclassification vary, closer attention needs to be paid to how samples are classified [62].

In the case considered, all sets within datasets were prepared as examples of binary classification with balanced classes. Both classes were considered as of the same importance, with the reasoning behind it that all text samples were expected and requested to be correctly attributed to their authors. In the case of studies of literary works, the misclassification costs were treated as equal for all classes. Therefore, the classification accuracy, obtained by dividing a number of correctly labelled samples by the total number of available samples, was the most natural to be employed as the performance measure, with intuitive interpretation [63,64].

Another factor to consider was how the process of testing was to be executed. Standard cross-validation approaches advocate the random selection of samples from a set, repeated a certain number of times, and averaging results over such folds [65]. However, in the case of stylometric input space, experiments show that random choice is not always the best way to go, even with increasing the number of folds above popular standards [66]. The problem lies in the specific distribution of datapoints in space, which is caused by the initial pre-processing of text samples. Random selection often results in choosing for tests such samples that are based on the same source texts that samples employed for training, which causes a leakage of information from learning onto testing and over-optimistic estimations of predictive powers. This problem can be solved by swapping groups of samples between train and test sets but at the cost of highly increased computational efforts. To avoid them, several separate test sets can be used, based on distinctively different source texts, over which averages are calculated. This last methodology was employed for all tests in the research described, as it provides a reasonable compromise between the reliability of the evaluation and the costs incurred. The element of randomisation was also ensured by the random selection of texts for train and test samples.

When rule sets are used to classify unknown samples, it is possible that several rules match a sample, but they disagree with respect to the assigned label, causing conflicts. In this situation, some strategy of resolving conflicts, such as voting, needs to be implemented. In the experiments, voting with weights was employed, giving each rule as many votes as its support. As a consequence, rules with higher supports, which are considered more important, had a greater influence on the final classification verdict.

## 4. Executed Experiments

The experiments performed included several, to some extent overlapping, paths, with most important aspects of these paths as follows:

- Sets of decision reducts were induced, either from numeric or discrete data;
- Based on sets of reducts, through the proposed weighting factor scores were assigned to variables and their rankings obtained;
- Sets of decision rules were inferred, either from continuous or categorical data;
- Elements of rule sets were pruned with the support of attribute rankings;
- Discretisation was treated as transformation applied not only to data but also to knowledge discovered in data and patterns captured by conditions included in decision rules.

All these elements and paths were presented and discussed in this section, with the summarising comments on the results at the end.

### 4.1. Reducts and Attribute Rankings

The ranking construction for the considered attributes always revolved around a specific set of generated decision reducts, based on which scores were obtained for the proposed weighting factor. The first set of reducts was obtained for data with continuous-valued attributes with DRSA processing. 4eMka software, employed in the research for reasoning with DRSA, offers the choice of either computing a single reduct, or all of them, and this latter task was executed, and for the two datasets, distinctively different numbers of reducts were found, just a few hundreds for F-writers and several thousands for M-writers. When these sets were processed, the rankings for attributes were returned, as shown in Table 1.

The score given for features assigns higher values, and by that higher ranking positions, to these attributes that were included in relatively more reducts, but at the same time taking into account reduct cardinalities, with the reasoning that smaller reducts are preferred as they offer higher reduction of dimensionality, which is possible only with strong features. Therefore, the attributes found in a core will always be high ranking, as can be seen for the comma for the F-writer dataset. To emphasise the fact that it is the only variable found in the core, it was separated by the vertical line in the table. On the other end, among the lowest ranking positions, the separation of *whether* from other features has the opposite meaning—this variable was absent in all reducts, so its score was equal to zero. When the rankings for the two datasets were compared, different positions given to the same variables confirmed previous expectations of observable contrasts between linguistic styles for writers of opposite sex, which was the basis for specific dataset construction.

The remaining rankings used in the research were built with calculations focused on discrete data, with the reduct generation algorithms implemented in the RSES system. The option of exhaustive search was considered, yet rejected, as finding all reducts for all 20 variants of discrete data per dataset would be very demanding. Furthermore, the initial tests performed indicated that the numbers of reducts found for this processing would be remarkably high, while the experiments show that it could be counterproductive, and rather smaller sets of reducts can be more effective [60]. Therefore, a genetic algorithm was employed in the search for reducts, with the input parameter of the requested number of reducts set to 200, as described in Section 3.4. With 20 sets of reducts found, as many rankings were obtained. For data processed with supervised discretisation by the methods of Fayyad and Irani and Kononenko, the rankings were given in Table 2.

Supervised discretisation can find some attributes useless from the point of view of their support for distinction of classes, and in fact for both methods and both datasets, such a situation occurred. It was indicated by separating some attributes at the bottom from the rest of the rankings. In a discrete domain, these variables had constant values for all samples; therefore, they were never included in the reducts and were assigned zero rank. In addition, they also never could appear in induced rules. As for the other features, interestingly, in this case, the comma was found as the highest ranking regardless of dataset and version of discretisation, while other variables had various ranks.

For unsupervised discretisation applied to the data, closer similarities were observed for the obtained rankings, as could be studied in Table 3 for equal frequency binning and in Table 4 for equal width binning. As several variants for both methods were obtained for the data, varying in the numbers of bins constructed (from 2 to 10), but with the same underlying transformations, these similarities were expected. Since not all reducts were generated, their intersection could not be called a core, and when a feature was not included in any of found reducts, it had lower significance than in case of availability of the entire sets of reducts, but different meaning than for total exclusion of some variable because of discretisation. In the former case, the variable had zero rank but could be encountered among conditions in inferred rules, while in the latter situation, they were included neither in reducts nor in rules.

Some attributes were included in all reducts in a considered set, and these were indicated at the top of the rankings, and some variables were missing. For the duw2 approach to discretisation, the cardinalities of reducts sets were considered differently because genetic algorithm found all reducts instead of their subset. In this case, the intersection of reducts was indeed a core, and it was non-empty at that.

Some attributes were repetitively found in a higher number of reducts, while others occurred again and again in just a few, and still for others, the positions taken strongly depended on the number of bins. For example, for M-writers in duf2 version, *any* is the lowest ranking feature, when for duf4, it took the second place from the top of the ranking. *On* belongs to the core for duw2, but it is placed in the middle of the ranking for duw8. Coincidentally (or not so much), for F-writers and duw2, *on* also belongs to the core, and for duw4, it was placed in the bottom half. The same could be observed for *until* in duf2 vs. duf4 version of the data.

Despite many similarities detected between the rankings obtained for discrete variants of the data, each ranking was different from others. All these rankings were then employed to guide the selection process of the attributes and decision rules. DRSA ranking (based on continuous data) was applied only to sets of decision rules with conditions on continuous values. The rest of the rankings (based on discrete data) were employed for DRSA rule sets with transformed conditions to make them discrete as well as for rule sets inferred by CRSA from discrete data, with matching methods of discretisation considered, which was described in the next section.

### 4.2. Ranking-Driven Rule Pruning

The process of rule pruning, explained in this section, was presented in three groups of obtained results, depending on the type of decision rules and their conditions:

- DRSA decision rules, induced from real-valued attributes, with conditions of continuous type (one set of rules per dataset);
- DRSA decision rules, inferred from numeric attributes, but with discretised conditions included in premises (one set of rules per dataset, but in 20 different versions, corresponding to 20 variants of discretisation approaches);
- CRSA decision rules, mined from discretised datasets so obviously with discrete conditions (20 different rule sets per dataset, each generated independently for each version of discrete dataset).

At the starting point of rule selection procedure, a full set of induced (and possibly transformed) decision rules was available as well as all condition attributes. For the sake of uniform presentation for all cases and all plots, the range of considered features is given from the overall maximum of 24 (all attributes taken under consideration) to the lowest possible minimum of 1 (a single feature left). This complete path allowed for a better observation of trends, although in practice, rule pruning would be stopped earlier if an unacceptable drop in performance would be detected. This performance was given as classification accuracy, a percentage of samples correctly recognised, with calculation of averages over test sets. In each case, the classification ratio for the complete set of attributes was treated as a reference point for the comparison of predictive powers for rule classifiers with fewer attributes and rules.

In the first step of rule pruning, from a considered ranking, the lowest ranking variable was excluded, and all decision rules with a condition on this rejected feature (regardless of other conditions being present or absent in the premise) were discarded as well. Then, the second step involved a reduction of the second worst attribute and all rules referring to it, and so on, for the next step, as long as there were some attributes left. It is possible that the rejection of some variable causes no change in the rule set when there are no rules with conditions on this feature (either they were removed in preceding steps because of conditions on other attributes or never existed in the first place). For some cases, it was possible to reach the final step of single remaining variables, but that depended on rule premises. If there were no rules with only one condition included, or if this condition did not match the feature that was kept, then at the end of processing, the rule set became empty. In the included charts, it was displayed as the classification accuracy equal to zero. The results close to zero correspond to cases when the numbers of rules were so low that they also provided very low coverage for test sets, causing poor recognition.

#### 4.2.1. DRSA Decision Rules

**Figure 2:**
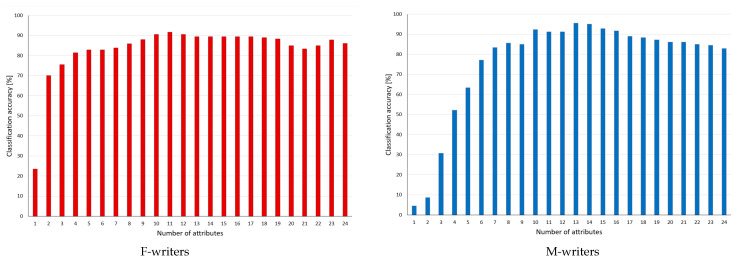
Classification accuracy observed in the process of pruning decision rules inferred from and with conditions on continuous values of attributes, with rankings based on reducts generated for continuous datasets. Categories for the X-axis indicate a number of attributes left in considerations.

The first group of tests involved decision algorithms working in the continuous domain, which referred to all 24 variables, and their reduction through rule pruning is shown in Figure 2. The chart displays the average performance for both datasets in a perspective of the number of features still included in considerations, which specify categories for the X-axis. For the complete set of features, the classification accuracy for F-writers was 86.12%, and for M-writers, it was 82.78%, but with rule pruning, these levels increased respectively to 91.67% for the 11 remaining variables and 95.56% for 13 features.

For the two datasets, similar trends could be noted—firstly some increase, in particular with the maximum performance around the middle, where roughly half of the attributes were left while the other half was rejected, and then decrease followed. The changes were steeper for the male writer dataset and gentler for the female writer dataset. In the case of F-writers, the performance became much degraded only at the very end, when there were just a few variables and rules available.

#### 4.2.2. DRSA Discretised Decision Rules

The second batch of experiments brought a discretisation of conditions in rules previously induced by DRSA data exploration, thus returning 20 variants of the same decision algorithm for each of the two datasets. Such a version was then matched with the corresponding ranking of attributes, which was constructed based on reducts found for discrete data. To emphasise the executed discretisation of real-valued conditions in rules, for all labels and references, the letter R was added before the abbreviated version of a name for the discretisation method commented.

**Figure 3:**
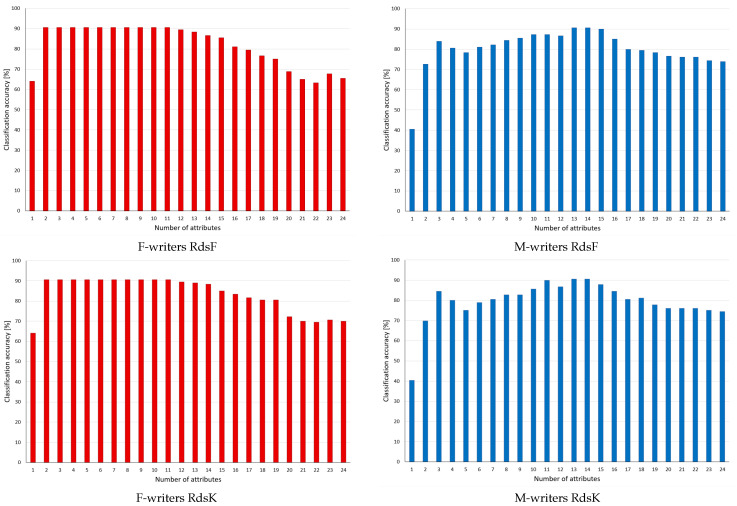
Classification accuracy observed in the process of pruning decision rules inferred from continuous values of attributes, whose values included in the conditions were discretised with supervised Fayyad and Irani (RdsF) and Kononenko (RdsK) methods, with rankings based on reducts generated for the corresponding discrete datasets. Categories for the X-axis indicate a number of attributes left in considerations.

In this group, the results for the supervised discretisation applied to the conditions of the rules are given in Figure 3. In the rankings considered here, some variables were assigned zero rank, as in the discrete domain, they were found as irrelevant and never included in reducts. However, the algorithms were induced from continuous data; thus, they referred also to these variables, and their rejection caused some rules to be discarded, which had some impact on performance.

For F-writers and both discretisation methods, it could be observed that after an initial gradual change to higher predictions, the performance levelled to be constant almost to the very end, when as few as just two variables were left, yet it was sufficient for enhanced classification. For M-writers, more variations in predictions could be found, yet here, improvement following rule pruning was also detected, with maximum again when around 50% of the variables were discarded.

**Figure 4:**
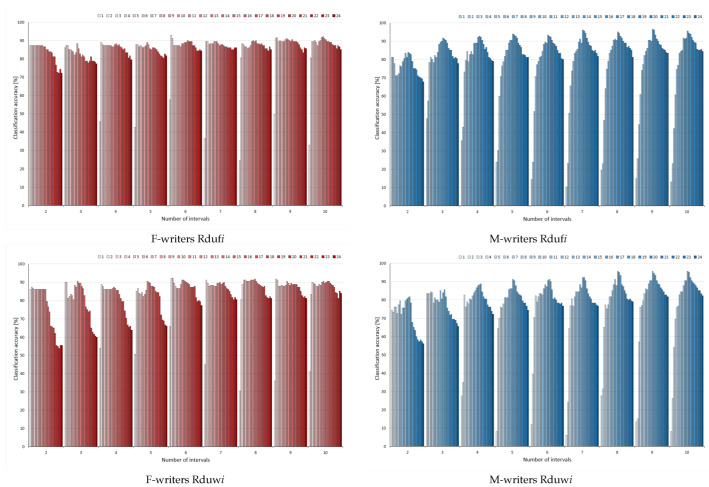
Classification accuracy observed in the process of pruning decision rules inferred from continuous values of attributes, whose values included in the conditions were discretised with unsupervised equal frequency (Rduf) method in the top row, and equal width (Rduw) method in the bottom row, with rankings based on reducts generated for matching discrete dataset. Categories for the X-axis indicate a number of intervals constructed for attributes in discretisation, while series correspond to the numbers of features left in considerations.

For unsupervised discretisation applied to conditions in rules, the results of rule pruning were shown in two perspectives. In the charts included in Figure 4, horizontal axes specify the number of bins constructed for attributes, while series correspond to the number of variables left in considerations. Then, each category shows the results obtained for a single rule filtering process, with steps of this process given by series (as before for Figure 2 and Figure 3, but in a more concise and condensed way). This form of presentation allowed observing similarities and differences in the process of rule pruning resulting from the particular value of the input discretisation parameter.

For female and male writer datasets for both discretisation methods, the worst results could be detected when only two intervals were constructed to represent the values of all considered attributes (Rduf2 and Rduw2), with the overall the lowest predictive powers of rule classifiers. To some extent, it could be concluded that higher bin numbers resulted in higher performance, which was best visible for the male writer dataset. However, when trends were compared between variants of a discretisation method, with different numbers of bins, they were very similar. From the starting level of classification for the full set of variables, along with gradual rule pruning, some enhanced predictions occurred, with the maximum detected either around the middle of each ranking or close to the top of it for F-writers. For M-writers, again, the initial increase was steeper and close to monotonic, but once some threshold of discarded rules was reached, the powers decreased rather rapidly and irrecoverably.

#### 4.2.3. CRSA Decision Rules

In the third path of experiments, the tests involved sets of rules induced from discrete versions of datasets, referring to the Classical Rough Set Approach. For each of the 20 variants of data prepared, with the exhaustive algorithm implemented in the RSES system, decision rules were inferred. Then, to each set, the process of rule pruning was applied, which was governed by the attribute ranking focused on reducts generated for this variant of data.

**Figure 5:**
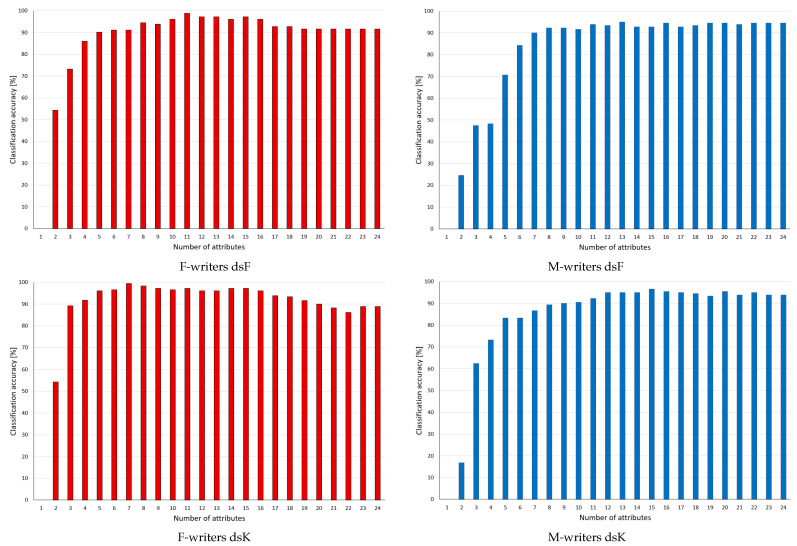
Classification accuracy observed in the process of pruning decision rules inferred from data discretised with supervised Fayyad and Irani (dsF) and Kononenko (dsK) methods, with rankings based on the reducts generated for the corresponding discrete datasets. Categories for the X-axis indicate a number of attributes left in considerations.

For the supervised discretisation approaches of Fayyad and Irani and Kononenko, the results obtained are given in Figure 5. As commented before, for both datasets, supervised discretisation caused some variables to be excluded from consideration because of assigning single bins for representation of their values. Consequently, these variables (indicated in the rankings in Table 2) occurred neither in reducts nor in rules. In the charts, this is visible through the unchanging level of predictions at the beginning phase of rule selection. Only after the lowest ranking variables present in the rules were discarded could some difference in performance be found.

As in general, rule sets based on discrete data performed better in classification than those induced earlier in the continuous domain, the changes were on a smaller scale, and not all of them were to advantage. In particular, at the end of processing, when only single features could still be included in the rules, no matching rules were found. However, in all four studied cases, enhanced performance was detected after discarding significant portions of attributes and rules from the considered sets.

**Figure 6:**
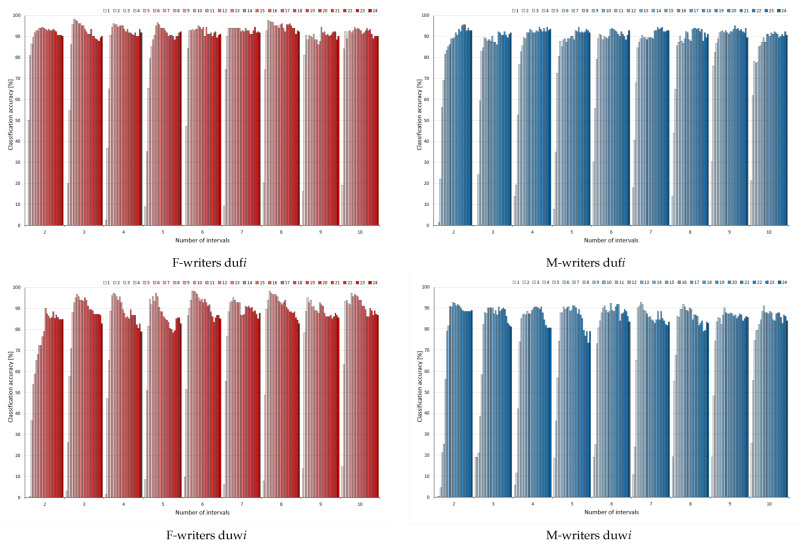
Classification accuracy observed in the process of pruning decision rules inferred from data discretised with unsupervised equal frequency (duf) method in the top row, and equal width (duw) method in the bottom row, with rankings based on reducts generated for the matching discrete dataset. Categories for the X-axis indicate a number of intervals constructed for attributes in discretisation, while series correspond to the numbers of features left in considerations.

Contrary to their rather popular bad reputation, unsupervised discretisation methods resulted in surprisingly high levels of predictions. In the charts shown in Figure 6, only the female writer dataset and equal width binning with 2 bins stands apart from the rest, with noticeably poorer performance. The remaining variants of discrete data enabled enhancing power at some point of rule selection; however, there was much more variety between the results, and some oscillations could be found.

Unlike for rules induced from continuous data, and then with the conditions discretised, for the rules inferred from discrete data, no particular dependence on a number of intervals could be found (with this single exception of duw2 for F-writers). The obtained maxima were rather close, or, if anything, some worsening of performance could be detected with more bins. For the male writer dataset, when some changes were found, the increase was not so steep as previously observed, and there were more better results for equal frequency binning than for the equal width for the same parameters (the same numbers of bins and attributes under considerations).

### 4.3. Summary of Results

An analysis of the charts with the performance of rule classifiers enabled concluding that in all paths of the experiments, some enhanced powers for inducers could be found and significant numbers of attributes and rules rejected, which validated the proposed methodology and showed the usefulness of reduct-based rankings. The process of rule pruning was shown for the entire possible range, starting with the complete set of attributes and rules, and with the stopping point when there was a single feature left with any rules referring to this feature. To further support the experimental results with some statistics, for all processed rule sets, the average performance and standard deviation per sample were calculated and provided in Table 5 and Table 6. To facilitate comparisons, also, performance was given for the complete rule sets; in both tables, it was listed in columns on the left.

From the range over which calculations were executed, the following steps were excluded: the starting point of all rules, and the end, for rules induced from continuous data, the last stage with single variables, and for rules inferred from discrete data the last two steps. For the former, the reasoning was to avoid influencing the results of rule pruning by the original performance with all rules. For the latter, the motivation was to obtain a clearer picture. Obviously, for most cases, for just one or two variables, if any, then only a few rules could still be included in a set, so performance was very low. However, cases of such reduction were rather extreme and artificially established, and they were given only for a complete view of the process and trends.

Table 5 presents the summary for the rule sets induced from the continuous data, and the letter R standing before the abbreviated name for a discretisation method indicates the discretisation applied to real-valued conditions in decision rules. For continuous conditions, the averaged performance for rule pruning in the observed range (from 23 to two attributes) was slightly lower than the reference point, and for M-writers, the standard deviation was high. However, if calculations were limited to the first half of discarded attributes (from 23 to 12), then the results were very different (88.85 ± 2.29 for F-writers, and 89.35 ± 3.81 for M-writers, respectively).

When the conditions in the rules were discretised, in the vast majority of cases, the rule reduction process led on average to higher predictions (at least slightly), with the exception of duf7, duf9, duf10, and duw10 for M-writers. The diversity of the classification results could be observed through the standard deviation—for the female writer dataset and unsupervised discretisation, *St.Dev.* decreased with increasing numbers of bins, while for the male writer dataset, the opposite trend could be detected.

Recapitulation of the results of the experiments in the case of the rule sets inferred from discrete data is given in Table 6. Here, the range over which statistics were calculated was set from 23 to 3 variables in the analysed set, since for fewer than that—for F-writers in 6 out of 20, and for M-writers in 5 out of 20 variants—there were no rules left. For these tests for the female writer dataset, only for two cases, duf5 and duw2, the average classification accuracy was lower then for the full set of rules. If the range was limited to from 23 to 12 attributes, for the former, still some decrease was present (but smaller) because only after these features were rejected, the performance obtained with the remaining rules started to increase. For duw2, the change in range brought the average of 85.79% and standard deviation of 2.68.

In the M-writer dataset, the situation was entirely different for equal frequency binning, where for all but one case (duf9), performance was degraded. However, when the range was limited once again to from 23 to 12 rejected features, either the decrease became only slight or changed to an increase. The same was true for duw2 and duw3, the only two versions for equal width binning, for which a worsening of averaged predictive powers was detected for the wider range of results taken under considerations.

It deserves to be pointed out that in all tests, the sets of rules used to classify unknown samples were employed with limitations on included rules coming only from the described process of rule pruning, without any other considerations, without taking into account rule quality measures, even these simplest, such as length or support. Imposing hard constraints on these elements, for example, keeping rules only with lengths smaller than, or with supports equal to at least some threshold, would most likely result in the overall higher predictive powers of rule classifiers, both for full sets of rules and their pruned subsets.

These condensed results further confirmed the soundness of the proposed research methodology and the effectiveness of decision reducts in their role as a source of knowledge on attributes that could be exploited for their weighting and ranking. However, when the process of rule pruning is executed, it needs to be closely controlled to avoid removing too many rules on one hand or not enough rules on the other. The question with respect to the stopping criterion is not easy to answer—the experiments show that a comparison of results between two subsequent steps does not provide sufficient information, as changes in performance are not necessarily exactly monotonic. To help with processing, the application of the evaluation and test sets would be recommended.

## 5. Conclusions and Future Research

The paper presents results of the research works on ranking-driven pruning of decision rules, inferred by rough set data exploration. To estimate the relevance and importance of the attributes, the proposed weighting factor was used, based on the notion of decision reduct, which is a mechanism dedicated to dimensionality reduction, inherent to the theory of rough sets. Decision reducts, which protect the predictive power of classification systems, were induced from data in the continuous domain with the Dominance-Based Rough Set Approach and also from data discretised by selected methods, with the Classical Rough Set Approach. The constructed rankings of features were used to control the rule pruning process for rule sets inferred with DRSA and with real-valued conditions, with discretised conditions, and for sets of rules induced by CRSA.

The experiments were carried out with stylometry as the application domain, which is focused on analysis of writing styles, with the main task of authorship attribution that was treated as a supervised learning problem. The characteristic features exploited referred to style markers, which allowed the description of stylistic profiles for authors. The chosen descriptors were of lexical and syntactic type, giving frequencies of occurrence for a set of function words and punctuation marks; consequently, the prepared datasets contained real values. The exploration of such data either requires inducers capable of operation in the continuous domain, or some discretisation approach needs to be implemented inside the data pre-processing step. In the research, three paths were tested: knowledge mining from real-valued attributes and then representation of discovered patterns also in such form, by conditions in rules being real-valued; inferring knowledge from continuous data but with discretisation of detected patterns, by discretisation of conditions included in rules; and finally, discretisation of datasets and then their mining.

The rankings that were obtained enabled observing and comparing the properties of decision reducts inferred from various forms of the same data. When they were employed for rule selection, also their usefulness for dimensionality reduction with respect to inducers was tested and proved to work to advantage, as long as attention was paid to the number of rules remaining in considerations and detected overall trends, as discarding both too few and too many rules can result in worsened performance. To help with establishing the stopping point for the pruning process, the usage of a standard approach with evaluation and test sets employed would be recommended.

One of the problems encountered in the experiments, which indicated one of the paths for future research, was the high computational complexity of all processes dedicated to decision rule induction. In the research, the rules were inferred from the input data in the continuous domain but also for all its discrete variants obtained through selected discretisation approaches. For all these cases, minimal cover algorithms returned so few rules that the classification of unknown samples brought disastrous results due to lack of coverage. The situation would be different if for testing, popular cross-validation could be used. Yet in stylometric input space, this method for evaluating performance gives unreliable over-optimistic estimation, making it unacceptable in standard version, even with higher numbers of folds and stratification. When test sets are used, more rules are required for good predictions, but the exhaustive search for rules for both DRSA and CRSA turned out to be very demanding. Therefore, one aim of future experiments was set to test other algorithms for rule induction. In addition, a limited number of algorithms were employed to infer decision reducts. When search procedures return only subsets instead of complete sets of elements, it stands to reason to expect various subsets with various search paths, as they can focus on other characteristic of the data. Since there are many algorithms for the induction of decision reducts, at least some of them could be explored.

Yet other path of future research leads to considerations on discretisation approaches beyond those most popular that were studied only with the limited range of parameters for unsupervised methods, with possible combination of methods for one dataset, depending on the properties of attributes. Finally, other application domains should be studied, in addition to stylometry. With different characteristics of the input data, a part of the processing could be simplified, for example, enabling standard cross-validation as a valid approach to estimate performance, but it could also lead to other observations. If all attributes were found to be of the same importance and assigned the same weighting score, the formulation of a ranking would be impossible, and the whole process would fail as a result. However, it is reasonable to expect such cases to be relatively rare.

## Figures and Tables

**Table 1:** Rankings of attributes based on DRSA decision reducts.

Rank	F-writers	M-writers
1	,	on
2	until	same
3	what	any
4	how	though
5	any	what
6	never	who
7	on	around
8	but	;
9	that	but
10	there	such
11	almost	by
12	before	how
13	within	,
14	though	before
15	same	never
16	after	whether
17	such	after
18	during	almost
19	who	then
20	around	within
21	;	there
22	by	that
23	then	until
24	whether	during

**Table 2:** Rankings of attributes based on decision reducts found in data after supervised discretisation by Fayyad and Irani (dsF) and Kononenko (dsK) approaches.

	dsF		dsK	
Rank	F-writers	M-writers	F-writers	M-writers
1	,	,	,	,
2	on	by	on	though
3	then	on	;	by
4	but	until	until	but
5	same	how	same	how
6	by	around	who	until
7	who	then	then	then
8	after	though	but	there
9	what	there	any	who
10	until	who	by	that
11	;	that	after	on
12	there	almost	within	around
13	any	;	before	;
14	before	such	what	what
15	around	any	though	any
16	such	same	such	same
17	never	what	never	almost
18	how	whether	there	before
19	whether	but	around	after
20	that	before	how	such
21	during	within	during	never
22	though	after	whether	within
23	almost	never	that	whether
24	within	during	almost	during

**Table 3:** Rankings of attributes based on decision reducts found in the data after the equal frequency unsupervised discretisation approach (duf*i*) with varying the number of intervals *i*.

	F-writers								
Rank	duf2	duf3	duf4	duf5	duf6	duf7	duf8	duf9	duf10
1	until	on	,	,	by	,	,	,	,
2	,	;	there	such	,	on	;	;	on
3	on	,	though	who	on	but	though	what	such
4	by	such	such	after	such	such	on	but	but
5	who	until	on	never	but	that	any	after	then
6	before	there	before	then	never	any	who	then	before
7	;	who	then	there	what	before	never	who	what
8	such	though	that	on	there	never	by	though	there
9	after	what	;	;	though	who	what	that	;
10	never	same	what	though	;	there	that	such	by
11	any	within	any	by	that	then	then	never	never
12	there	then	by	how	how	after	until	before	same
13	within	but	how	but	before	;	such	on	who
14	but	any	but	any	who	what	before	how	after
15	around	that	after	that	any	by	but	by	how
16	then	before	who	what	same	within	there	there	until
17	how	never	until	same	after	how	how	within	though
18	what	by	almost	until	then	though	same	same	that
19	same	almost	around	around	around	same	after	any	any
20	that	how	never	within	until	until	almost	until	within
21	almost	during	within	almost	almost	almost	within	almost	around
22	though	after	same	before	within	around	around	whether	almost
23	whether	around	during	whether	during	during	during	around	whether
24	during	whether	whether	during	whether	whether	whether	during	during
	**M-writers**								
1	,	there	,	,	,	,	,	,	,
2	by	by	any	until	by	after	what	by	there
3	until	though	there	there	there	any	before	that	who
4	same	any	then	but	on	on	on	after	after
5	then	,	until	;	until	same	but	how	that
6	what	but	though	almost	then	never	how	on	by
7	there	that	by	how	almost	until	who	;	same
8	almost	after	who	what	never	that	after	before	any
9	that	until	on	before	before	then	until	but	what
10	though	same	same	any	but	before	;	though	almost
11	around	how	what	such	that	there	there	never	but
12	how	then	how	by	how	how	then	almost	around
13	but	who	never	then	same	what	never	what	;
14	on	what	but	same	who	who	by	until	until
15	never	;	;	on	such	by	around	then	on
16	such	on	almost	that	though	almost	same	there	how
17	before	such	before	though	any	;	that	same	never
18	;	never	after	after	around	such	almost	such	before
19	after	before	such	who	;	around	though	any	then
20	whether	almost	that	never	what	but	any	who	though
21	during	within	during	around	after	though	during	around	such
22	within	during	around	within	whether	during	such	during	during
23	who	around	whether	whether	within	within	whether	within	within
24	any	whether	within	during	during	whether	within	whether	whether

**Table 4:** Rankings of attributes based on decision reducts found in the data after the equal width unsupervised discretisation approach (duw*i*) with varying the number of intervals *i*.

	F-writers								
Rank	duw2	duw3	duw4	duw5	duw6	duw7	duw8	duw9	duw10
1	on	,	,	,	,	,	,	,	,
2	but	;	;	;	;	before	any	;	though
3	never	on	how	there	though	there	;	there	;
4	,	never	though	never	who	almost	such	then	that
5	;	any	there	that	such	until	on	but	such
6	same	but	within	how	on	though	but	any	within
7	what	until	until	then	that	never	before	how	on
8	almost	that	before	on	then	how	that	that	any
9	that	then	such	any	what	that	until	by	never
10	how	same	any	but	by	who	who	after	before
11	such	almost	by	before	any	what	though	before	what
12	though	before	but	though	until	such	after	on	but
13	any	there	who	such	never	;	almost	though	there
14	until	what	then	same	there	by	same	never	how
15	before	though	what	by	but	on	what	such	then
16	within	how	that	after	after	then	then	who	after
17	by	after	on	who	same	but	there	same	who
18	after	who	never	what	almost	same	how	what	same
19	then	around	after	almost	around	any	within	until	until
20	there	within	same	around	how	after	never	almost	by
21	who	such	almost	until	before	within	by	within	almost
22	whether	by	whether	within	within	during	around	around	around
23	around	whether	around	whether	whether	whether	during	during	whether
24	during	during	during	during	during	around	whether	whether	during
	**M-writers**								
1	on	,	,	,	,	,	,	,	,
2	same	then	until	by	but	never	by	by	then
3	,	after	but	there	by	by	then	that	before
4	but	on	there	what	almost	on	that	then	that
5	there	any	who	on	who	that	there	until	who
6	that	there	almost	then	until	what	around	almost	but
7	then	but	any	until	on	but	but	before	on
8	never	what	that	almost	around	then	never	on	there
9	what	who	on	after	before	who	almost	there	by
10	by	by	then	but	then	though	before	after	how
11	until	until	though	before	how	after	;	who	same
12	after	that	by	that	there	there	on	same	;
13	almost	almost	never	how	that	before	any	how	until
14	who	how	what	who	what	around	how	though	such
15	how	around	after	any	;	any	until	what	though
16	any	never	before	such	within	almost	what	;	after
17	before	within	within	though	after	until	after	never	almost
18	around	though	same	;	during	how	though	but	what
19	whether	before	such	never	any	within	who	such	any
20	such	;	whether	around	though	;	during	any	around
21	;	whether	around	same	such	whether	whether	during	never
22	during	such	how	during	same	same	same	around	within
23	within	same	;	whether	never	such	within	within	during
24	though	during	during	within	whether	during	such	whether	whether

**Table 5:** Performance (%) for rule classifiers with the complete sets of rules, and for the process of rule pruning for rule sets induced from continuous data by DRSA (classification accuracy for full rule sets and average classification accuracy ± standard deviation for subsets).

		Full Sets of Rules Available		Subsets of Rules Returned by Pruning	
		Dataset		Dataset	
		F-writers	M-writers	F-writers	M-writers
Real		86.16	82.78	85.85 ± 05.11	79.15 ± 21.46
RdsF		65.56	73.89	83.31 ± 09.34	82.14 ± 05.23
RdsK		70.00	74.45	84.80 ± 07.53	81.48 ± 05.52
	2	72.22	67.78	83.53 ± 04.92	76.57 ± 04.77
	3	77.23	77.78	82.32 ± 03.14	83.29 ± 07.09
	4	79.45	78.89	86.21 ± 02.11	83.15 ± 10.04
	5	81.67	81.11	85.41 ± 02.28	81.89 ± 13.72
Rduf	6	84.45	80.00	87.90 ± 02.00	80.98 ± 15.30
	7	86.12	81.67	87.55 ± 01.41	81.59 ± 16.17
	8	85.00	81.11	87.15 ± 02.07	81.90 ± 16.72
	9	85.56	83.34	89.02 ± 02.06	81.37 ± 16.71
	10	85.00	84.45	88.58 ± 02.41	81.39 ± 17.43
	2	55.56	56.12	75.33 ± 12.52	71.21 ± 08.45
	3	60.00	65.56	79.65 ± 09.50	78.11 ± 05.86
	4	63.89	72.22	81.44 ± 07.49	79.29 ± 10.55
	5	66.12	74.45	82.75 ± 06.78	80.90 ± 06.15
Rduw	6	77.23	76.67	87.35 ± 03.81	80.70 ± 10.23
	7	80.56	76.67	87.22 ± 02.91	79.62 ± 13.52
	8	81.11	78.89	88.00 ± 03.68	81.35 ± 12.93
	9	81.11	81.67	87.73 ± 02.58	81.96 ± 16.58
	10	83.89	82.22	87.91 ± 02.59	81.92 ± 15.09

**Table 6:** Performance (%) for rule classifiers with the complete sets of rules, and for the process of rule pruning for rule sets induced from discrete data by CRSA (classification accuracy for full rule sets and average classification accuracy ± standard deviation for subsets).

		Full Sets of Rules Available		Subsets of Rules Returned by Pruning	
		Dataset		Dataset	
		F-writers	M-writers	F-writers	M-writers
dsF		91.67	94.45	92.51 ± 05.25	87.47 ± 13.84
dsK		88.89	93.89	94.18 ± 03.73	89.98 ± 08.27
	2	90.00	92.78	91.68 ± 02.30	80.24 ± 24.27
	3	90.00	91.67	90.83 ± 08.82	84.39 ± 15.05
	4	91.67	93.33	91.70 ± 06.27	88.90 ± 09.12
	5	92.23	91.67	89.67 ± 06.57	89.04 ± 04.82
duf	6	91.11	92.78	92.01 ± 02.30	90.24 ± 02.98
	7	91.67	92.78	92.91 ± 01.16	89.64 ± 05.47
	8	92.23	91.12	94.84 ± 01.67	89.23 ± 06.07
	9	90.00	89.45	90.38 ± 01.88	91.70 ± 02.70
	10	90.00	90.56	92.09 ± 01.55	88.12 ± 04.67
	2	84.69	88.89	73.06 ± 21.02	72.79 ± 30.44
	3	82.78	81.11	88.62 ± 08.76	80.79 ± 18.05
	4	78.89	80.56	88.13 ± 07.09	84.26 ± 10.26
	5	82.78	78.89	87.14 ± 05.65	84.44 ± 08.31
duw	6	85.00	83.34	91.61 ± 04.45	87.50 ± 04.26
	7	87.78	83.34	89.61 ± 03.95	85.53 ± 05.52
	8	82.78	82.78	91.99 ± 03.94	85.38 ± 05.29
	9	85.56	85.56	89.11 ± 02.86	85.58 ± 03.02
	10	86.67	83.89	91.70 ± 03.48	85.27 ± 03.76

## Data Availability

Data available upon request.
